# The Relationship between Side of Onset and Cerebral Regional Homogeneity in Parkinson's Disease: A Resting-State fMRI Study

**DOI:** 10.1155/2020/5146253

**Published:** 2020-06-27

**Authors:** Kai Li, Hong Zhao, Chun-Mei Li, Xin-Xin Ma, Min Chen, Shu-Hua Li, Rui Wang, Bao-Hui Lou, Hai-Bo Chen, Wen Su

**Affiliations:** ^1^Department of Neurology, Beijing Hospital, National Center of Gerontology, No. 1 Dahua Road, Dong Dan, Beijing 100730, China; ^2^Department of Radiology, Beijing Hospital, National Center of Gerontology, No. 1 Dahua Road, Dong Dan, Beijing 100730, China

## Abstract

**Objective:**

Motor symptoms are usually asymmetric in Parkinson's disease (PD), and asymmetry in PD may involve widespread brain areas. We sought to evaluate the effect of asymmetry on the whole brain spontaneous activity using the measure regional homogeneity (ReHo) through resting-state functional MRI.

**Methods:**

We recruited 30 PD patients with left onset (LPD), 27 with right side (RPD), and 32 controls with satisfactory data. Their demographic, clinical, and neuropsychological information were obtained. Resting-state functional MRI was performed, and ReHo was used to determine the brain activity. ANCOVA was utilized to analyze between-group differences in ReHo and the associations between abnormal ReHo, and various clinical and neuropsychological variables were explored by Spearman's correlation.

**Results:**

LPD patients had higher ReHo in the right temporal pole than the controls. RPD patients had increased ReHo in the right temporal pole and decreased ReHo in the primary motor cortex and premotor area, compared with the controls. Directly comparing LPD and RPD patients did not show a significant difference in ReHo. ReHo of the right temporal pole was significantly correlated with depression and anxiety in RPD patients.

**Conclusions:**

Both LPD and RPD have increased brain activity synchronization in the right temporal pole, and only RPD has decreased brain activity synchronization in the right frontal motor areas. The changed brain activity in the right temporal pole may play a compensatory role for depression and anxiety in PD, and the altered cerebral function in the right frontal motor area in RPD may represent the reorganization of the motor system in RPD.

## 1. Introduction

Parkinson's disease (PD) is the second most common neurodegenerative disorder. It is well known that PD has motor symptoms including bradykinesia, resting tremor, and rigidity, as well as various nonmotor symptoms [1]. Most of the PD patients initially present unilateral motor symptoms, and this motor symptom asymmetry persists afterwards [2, 3]. This feature is unique and can help differential diagnosis from atypical Parkinsonism [4]. Although unequal degeneration of dopaminergic neurons in the midbrain can interpret this motor asymmetry [5–7], the influence of lateralization is widespread and involves a variety of aspects of PD.

Lateralization modulates multiple nonmotor symptoms, including cognitive impairment, anxiety, apathy, psychosis, rapid eye movement sleep behavior disorder, and olfactory dysfunction [8–13]. PD patients with left onset (LPD) and right onset (RPD) respond differently to levodopa and rehabilitation treatments in some cognitive domains [14, 15]. Furthermore, LPD and RPD may have different disease progression speed and risk of motor complications [9, 16, 17]. Why lateralization has such extensive effects remains unclear. Uneven disturbance of bilateral corticostriatal -thalamic circuits might partially account for this phenomenon; lateralized neurodegeneration in multiple brain areas may also have contributions [8, 12]. Additionally, hemispheric dominance may interact with lateralization of neurodegeneration and play a role [18].

Magnetic resonance imaging (MRI) is a noninvasive modality, and it can be used to explore the effects of lateralization on the brain structure and function, and thus, it helps us understand the underlying mechanism and further implement individualized treatment. The hemisphere contralateral to the side of onset has a greater atrophy than the hemisphere ipsilateral to the side of onset [19]. Moreover, the volume of the lateral ventricular contralateral to the side of onset has a faster rate of enlargement than the ipsilateral side, which indicates accelerated neurodegeneration of the hemisphere contralateral to the side of onset [20].

Functional MRI (fMRI) assesses the blood oxygen level dependent (BOLD) effect in the brain, which represents cerebral blood flow and energy use [21]. fMRI is a valuable tool to measure the neuronal activity, either during specific tasks or under a resting-state (rs-fMRI). The former requires the participant to perform a specific task, and the later needs almost no effort from the subject [21]. Task-based fMRI have demonstrated that LPD and RPD had different compensatory brain activities during passive movements [22]. Various approaches have been employed in the analysis of rs-fMRI data, including methods for the local activity across the whole brain (such as regional homogeneity (ReHo)) and procedures exploring the relationship between different brain regions (such as functional connectivity) [23]. However, only few studies utilized rs-fMRI to examine spontaneous brain activities in LPD and RPD separately. Tang and colleagues used the functional connectivity analysis and found that compared with LPD and controls, RPD patients had an aberrant functional connectivity in the brain areas of the left somatosensory and motor networks, as well as the default mode network (DMN) [23]. Huang et al. measured ReHo in the striatum and found decreased ReHo in the right dorsal rostral putamen of LPD patients, compared with RPD patients and normal controls. Their results confirmed the asymmetry of basal ganglia function in PD [24]. ReHo measures the functional similarity between a voxel and its neighbour voxels. It is a data-driven approach and can conveniently evaluate the function of the whole brain [25]. To date, it is unknown how the brain activity synchronization changes in LPD and RPD patients. We assume that motor symptom asymmetry can affect brain function synchronization in multiple areas in PD and might interact with hemispheric dominance. Therefore, the present study aimed to assess the influence of lateralization on brain activities in PD using ReHo.

## 2. Materials and Methods

### 2.1. Participants

Sixty-three patients with PD and 33 age and sex-matched healthy controls with no history of neurological or psychiatric disorders were recruited from Beijing Hospital between 2012 and 2014. All the patients were diagnosed by an neurologist with expertise in Parkinson's disease, based on the United Kingdom Parkinson's Disease Society brain bank diagnostic criteria [4].

Clinical evaluations, including medical history and physical and neurological examinations were performed in all the subjects. All the subjects were right handed. Side of the motor symptom onset was identified by medical records and patients' reports and was supported by neurological examination. The patients were excluded if the side of onset could not be ascertained consistently or with bilateral onset. We excluded PD patients with dementia, moderate to severe head tremor, head trauma, deep brain stimulation, alcohol or drug abuse, or with other neurological or psychiatric disorders.

MRI examination, motor and nonmotor function assessments were performed after withdrawing all the anti-Parkinsonian medications for ～12 h. Unified PD Rating Scale (UPDRS) part III, Hoehn–Yahr staging, Hamilton Depression Rating Scale (HAMD), Hamilton Anxiety Rating Scale (HAMA), and Nonmotor Symptoms Questionnaire (NMSQ) were evaluated in all the PD patients.

The study was approved by the Ethics Committee of Beijing Hospital and carried out according to the Declaration of Helsinki. Written informed consent was given by all the participants.

### 2.2. Image Acquisition

MRI images were acquired on a 3.0 Tesla MRI scanner (Achieva TX, Philips Medical Systems, Best, Netherlands) at Beijing Hospital. Tight foam padding was employed to reduce head movement, and headphones were used to minimize scanning noise. The subjects were required to relax with their eyes closed and remain awake. High resolution 3D T1-weighted images were obtained with the following parameters: repetition time (TR) = 7.4 ms, echo time (TE) = 3.0 ms, flip angle (FA) = 8°, field of view (FOV) = 240 × 240 mm, matrix size = 256 × 256, voxel dimensions = 0.94 × 0.94 × 1.20 mm, and slice thickness = 1.2 mm, 140 slices. rs-fMRI data were collected axially using echo-planar imaging (EPI) with the following parameters: TR = 3000 ms, TE = 35 ms, flip angle = 90°, FOV = 240 × 240 mm, matrix size = 64 × 64, voxel dimensions 3.75 × 3.75 × 4.00 mm, slice thickness = 4 mm, slices = 33, and time points = 210.

### 2.3. Rs-fMRI Data Preprocessing

rs-fMRI data preprocessing was conducted using RESTPlus V 1.2 [26], based on SPM 12 (http://www.fil.ion.ucl.ac.uk/spm). The first 10 volumes were discarded for magnetization stabilization and subjects' adaptation. Then, the following steps were included: slice-timing, realignment to account for head motion, spatial normalization to the Montreal Neurological Institute (MNI) template using the coregistered T1 images (by DARTEL) [27], resampling to a resolution of 3 × 3 × 3 mm^3^, time course detrending, nuisance covariates regression (Friston-24 head motion parameters [28], white matter, and cerebrospinal fluid signals), and band-pass filtering (0.01*f* < 0.1 Hz). Participants with head motion exceeding 2 mm in displacement or 2° in rotation were excluded.

### 2.4. ReHo Calculations

ReHo maps were generated using RESTPlus V 1.2, with the procedures published previously [29]. Kendall's coefficient of concordance (KCC) was calculated between the time series of each voxel and its nearest 26 neighbour voxels in a voxel-wise way across the whole brain. For standardization purposes, KCC of each voxel was divided by the average KCC of the whole brain to obtain normalized ReHo maps. Finally, ReHo maps were smoothed using a Gaussian kernel (6 mm full-width-half-maximum, FWHM).

### 2.5. Statistical Analysis

SPSS (Version 23.0. Armonk, NY : IBM Corp) was used for the analysis of clinical information. The quantitative data are presented as mean ± standard deviation. Data normality was evaluated by the Kolmogorov–Smirnov test. One-way ANOVA, the Kruskal–Wallis test, *t*-test, or Mann–Whitney *U* test was utilized to compare numerical variables between the LPD, RPD, and control groups when applicable. Chi-square or Fisher's exact test was employed for comparisons of categorical variables. *P* < 0.05 was considered statistically significant.

With the help of DPABI V4.2 [30], between-group differences of ReHo were analyzed using ANCOVA with age and grey matter density as covariates. The grey matter mask in DPABI V4.2 was used. The LSD method was utilized for post hoc pairwise analyses. The resultant *T* maps were corrected based on the Gaussian random field theory (GRF) (voxel-level *P* < 0.001; cluster-level *P* < 0.05; two-tailed) [31, 32]. Averaged ReHo values of clusters with significant between-group differences were extracted and correlated with clinical and neuropsychological variables via Spearman's correlation.

To explore the potential interaction between hemispheric dominance and motor asymmetry in PD, we performed paired *t*-tests between ReHo of the left and right hemispheres in the LPD and RPD groups, with grey matter density as a covariate. Then, we conducted a mixed effect analysis, which included two groups (LPD and RPD) and two conditions (dominant and nondominant hemispheres), with grey matter density and age as covariates. In addition, we used the image calculator tool of DPABI to generate between-hemispheric ReHo difference image files by subtracting the ReHo map of the right hemisphere from the ReHo map of the left hemisphere. Afterwards, we compared the between-hemispheric ReHo difference images between the LPD and RPD patients using the *t*-test.

## 3. Results

### 3.1. Clinical Features

We excluded 5 PD patients and 1 control subject due to excessive head motion. One PD patient was excluded because of unsatisfactory image quality. Ultimately, we included 57 patients with PD and 32 controls. Thirty PD patients initially presented motor symptom in the left side and 27 in the right side.

The demographic and clinical data are shown in Table 1. No significant difference was found in age or sex among the three groups. Disease duration was comparable in the LPD and RPD groups. There was no significant difference in UPDRS, Hoehn–Yahr staging, HAMD, HAMA, or NMSQ scores between the LPD and RPD groups.

### 3.2. Group Differences in ReHo

ANCOVA and post hoc pairwise analyses revealed significant differences in ReHo between the two PD groups and the control group, while there was no significant difference between the LPD and RPD groups.

**Figure 1:**
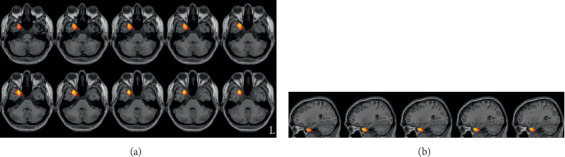
The ReHo difference between the LPD group and the control group. (a) and (b) are axial and sagittal views, respectively. LPD patients had increased ReHo in the right temporal pole compared with the controls. L indicates the left side.

**Figure 2:**
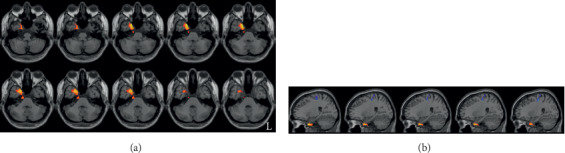
Increased ReHo in the right temporal pole in RPD patients compared to the controls. (a) and (b) are axial and sagittal views, respectively. L indicates the left side.

**Figure 3:**
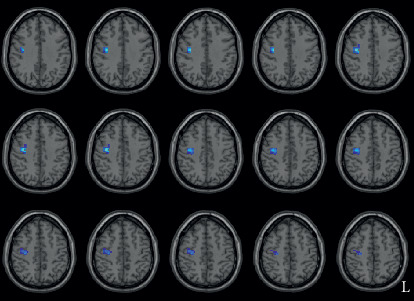
Decreased ReHo in the right precentral gyrus and right middle frontal gyrus. L indicates the left side.

LPD patients had increased ReHo in the right temporal pole, compared with the controls (Figure 1 and Table 2). RPD patients also showed higher ReHo in the right temporal pole, and they additionally showed lower ReHo in the right precentral gyrus and right middle frontal gyrus. The results are shown in Figures 2 and 3 and Table 2. To further evaluate the difference of ReHo in the right frontal lobe cluster between LPD and RPD patients, we extracted the ReHo value of this region and performed a Bayesian estimation using an online tool (http://sumsar.net/best_online/). The result showed that the 95% highest density interval did not include 0.

**Figure 4:**
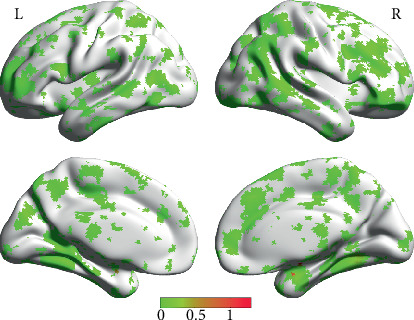
Effect sizes (Cohen's ƒ^2^) of between-group ReHo differences (LPD vs. RPD). Cohen's ƒ^2^ was thresholded at higher than 0.02. L indicates the left side, and R indicates the right side.

Although directly comparing the LPD and RPD groups did not obtain positive results, it might be helpful to know the effect size of between group comparisons.  Figure 4 illustrates the effect sizes (Cohen's ƒ^2^) of between-group differences (LPD vs. RPD). Cohen's ƒ^2^ was thresholded at higher than 0.02, which is the lower limit of a small effect [33].

### 3.3. Correlation Analysis

Spearman' correlation was carried out to explore the associations between changed ReHo and Hoehn–Yahr staging, UPDRS part III, HAMD, HAMA, and NMSQ scores. ReHo of the positive cluster in the right temporal pole was significantly correlated with HAMD, HAMA, and NMSQ scores (*r*  = −0.485, −0.442, and −0.398; *P* = 0.011, 0.021, and 0.040, respectively) in the RPD group. No significant correlation was found between ReHo of the right frontal cluster and the clinical or neuropsychological variables in the RPD group. In the LPD group, there was no significant association between the changed ReHo and the clinical or neuropsychological indices.

### 3.4. The Interaction between Hemispheric Dominance and Motor Asymmetry

First, comparing bilateral hemispheres in the LPD and RPD groups obtained generally similar results. In both groups, regions with significant differences were mainly located at the medial parietal lobe, the medial occipital lobe, the posterior cingulate cortex, the superior temporal lobe, and insula. There was an exception that there was one positive cluster in the anterior cingulate cortex only in the RPD group.

The mixed effect analysis showed no interaction between hemispheric dominance and motor symptom laterality. The comparison of between-hemisphere ReHo difference images between the LPD and RPD patients also found no cluster with significant between-group difference.

## 4. Discussion

As far as we know, this is the first study showing different patterns of aberrant spontaneous brain activities in LPD and RPD patients, with the help of ReHo. We found that both LPD and RPD patients exhibited increased ReHo in the right temporal pole; moreover, RPD patients showed decreased ReHo in the right precentral gyrus and right middle frontal gyrus. Furthermore, ReHo in the right temporal pole was associated with nonmotor symptoms in PD, especially depression and anxiety.

In the present study, both LPD and RPD patients displayed increased ReHo in the right temporal pole. Previous studies have also identified this area with abnormal ReHo in PD, but its clinical significance in PD has rarely been discussed [34–37]. Although the two groups had different patterns of asymmetry of motor symptoms, most of the patients had bilateral motor symptoms. Even in the patients with unilateral symptoms, they already have bilateral neurodegeneration in the basal ganglia [38]. Therefore, it is unsurprising that LPD and RPD patients have some overlapping changes in the brain activity.

In addition to increased ReHo in the right temporal pole, we further demonstrated significant negative correlations between this changed ReHo and nonmotor symptoms, especially depression and anxiety, which indicated that the increased neuronal synchronization in this region might be a compensatory mechanism for depression and anxiety. It is already known that depression and anxiety are common nonmotor symptoms in PD [39, 40]. Depression and anxiety in PD mainly arise from neurodegeneration in multiple nuclei in the brain stem and are associated with several neurotransmitter abnormalities (encompassing serotonin, noradrenaline, dopamine, and GABA) [39, 40]. In addition, several cortex areas are also involved [41]. Temporal pole has close connections with key structures related with emotion, such as amygdala, orbital frontal cortex, prefrontal cortex, basal forebrain, and hypothalamus. Particularly, the right temporal pole plays a critical role in the regulation of emotion and has been demonstrated to be related with major depression and anxiety disorders, as well as depression and anxiety symptoms in various diseases [42–44]. In Parkinson's disease, nuclei linked to depression and anxiety in the brain stem (especially the raphe nuclei and locus coeruleus) are compromised at the earliest stages, when the cerebral cortices are spared [45]. Increased ReHo indicates enhanced local synchronization and may reflect neural hyperactivity [46]. Therefore, a relatively spared right temporal pole has an enhanced neural activity to compensate for the disrupted function of compromised brain stem nuclei, in order to relieve the depression and anxiety symptoms in PD. Our results shed new light on the compensation mechanism of anxiety and depression in PD, which emphasize the role of the right temporal pole.

In addition to the similar alteration of ReHo in the right temporal lobe in the two PD groups, we found decreased ReHo in the right primary motor cortex and premotor area only in RPD patients. Decreased ReHo in this region has been reported in several studies including a meta-analysis [47–50]. It is to be noted that LPD and RPD patients were combined as a single group in these studies. Based on our results, we infer that RPD patients might make a major contribution to the similar group differences in these previous studies. It is recognized that RPD patients have more severe neurodegeneration in the left substantia nigra, which causes a larger influence in the left corticostriatal -thalamic circuit [5, 6, 51]. However, our results showed abnormal ReHo in the right hemisphere, and this may be due to the reorganization of the motor symptom in PD. Two task-based fMRI studies used unilateral hand movement paradigms, and they found an increased activity of the right frontal motor area in RPD patients when they were using their right hand, compared with the control subjects [22, 52]. The hyperactivation of the ipsilateral hemisphere may represent a compensatory mechanism for the dysfunction of the contralateral corticostriatal -thalamic circuit [22]. Thus, our study corroborates the phenomenon of the abnormal brain activity in the frontal motor cortex ipsilateral to the side of onset using rs-fMRI, which may play a compensatory role. The decrease of resting ReHo in our study is not contradictory to the increased brain activity of the same area in the studies using task-based fMRI, as the pattern of the brain activity usually differs between the resting and task states [53]. The present study underscores the necessity of separating LPD and RPD patients when studying the brain activities of PD.

Although the Bayesian estimation and 95% confidence interval indicated that the ReHo of the frontal cluster in Table 2 might differ between the two PD groups, this cluster did not survive our multiple comparison correction. In addition, we computed the effect sizes of difference of ReHo between the LPD and RPD patients, and almost all the clusters shown in Figure 4 belonged to small effect size. These results indicate that the LPD and RPD patients might only have minor differences in the brain activity, which warrant further investigation. Interestingly, Pelizzari and colleagues employed a similar study paradigm using diffusion-weighted magnetic resonance imaging. They only identified significant difference of mean diffusivity between the RPD and the control groups but not between the RPD and the LPD groups or between the LPD and the control groups [54]. Considering their study and the present research, RPD patients might have different changes in the brain structure and activity from LPD patients.

There are three limitations of this study. First, the sample size is not large. We only identified different spontaneous brain activities between the two PD groups and the controls. The relatively small sample size may account for the lack of significant ReHo difference from the direct comparison between the two PD groups. Future studies need to include more patients to better clarify the feature of brain activities in LPD and RPD patients. Second, most of the patients had bilateral symptoms in the present study, and thus, the impact of lateralization might be minor and difficult to detect. Further studies recruiting more patients and particularly those with only unilateral symptoms may better disclose the influence of motor asymmetry on cerebral activities. Third, although we evaluated the PD patients during an off period to reduce the pharmacological effects, the influence of the anti-Parkinsonian medications cannot be completely ruled out. However, this is a commonly used strategy and help comparisons between our study and similar studies from other researchers. In addition, one study observed similar alterations of ReHo in de novo PD patients and off-medication patients [50]. Therefore, the effects of anti-Parkinsonian medications should not be a major concern.

In conclusion, we found that both LPD and RPD patients have increased ReHo in the right temporal pole in comparison to healthy controls, and only RPD patients have decreased ReHo in the right frontal motor area. These results indicate that the right temporal pole plays a compensatory role for depression and anxiety in PD and reflect the reorganization of the motor system in PD. These results stress the importance to study the similarity and difference between LPD and RPD patients in future studies using functional imaging modalities.

## Figures and Tables

**Table 1:** Demographic and clinical characteristics of the PD patients and controls.

	LPD	RPD	Controls	*P* value
Number of subjects	30	27	32	
Age	62.63 ± 8.88	65.85 ± 6.982	62.41 ± 7.07	0.056
Gender (male/female)	14/16	14/13	16/16	0.924
Disease duration	6.80 ± 3.62	6.15 ± 3.59		0.499
Hoehn–Yahr staging	2.13 ± 0.71	2.28 ± 0.67		0.416
UPDRS III	30.90 ± 12.59	29.67 ± 19.09		0.676
HAMD	9.07 ± 5.27	9.56 ± 5.09		0.724
HAMA	9.93 ± 5.04	10.52 ± 6.03		0.691
NMSQ	11.07 ± 5.77	11.56 ± 4.86		0.732

HAMA, Hamilton Anxiety Rating Scale; HAMD, Hamilton Depression Rating Scale; LPD, Parkinson's disease with left onset; RPD, Parkinson's disease with right onset; NMSQ, Nonmotor Symptoms Questionnaire; and UPDRS, Unified Parkinson's Disease Rating Scale.

**Table 2:** Brain regions with significant differences in ReHo between PD patients and control subjects.

Brain regions	Side	Peak MNI coordinates			Number of voxels	*T*-value	Effect size (Cohen's ƒ^2^) of LPD vs. RPD	95% Confidence interval of LPD vs. RPD
		*X*	*Y*	*Z*				
LPD > HC								
Right temporal pole	R	30	12	−36	130	5.30	0.038	−0.037, 0.137
RPD > HC								
Right temporal pole	R	21	9	−42	90	4.25	0.013	−0.088, 0.101
RPD < HC								
Right precentral gyrus and right middle frontal gyrus	R	36	−12	42	161	−5.49	0.06	0.016, 0.098

HC, healthy controls; LPD, Parkinson's disease with left onset; MNI, Montreal Neurological Institute; *R*, right; and RPD, Parkinson's disease with right onset.

## Data Availability

The data supporting the findings of this study are available from the corresponding authors upon request.
